# Influence of Selective Dopamine Agonist Ropinirole on Conditioned Place Preference and Somatic Signs of Morphine Withdrawal in Rats

**DOI:** 10.3389/fnbeh.2022.855241

**Published:** 2022-06-06

**Authors:** Andleeb Shahzadi, Oruc Yunusoglu, Enes Karabulut, Haktan Sonmez, Zeliha Yazici

**Affiliations:** ^1^Department of Medical Pharmacology, Faculty of Medicine-Cerrahpasa, Istanbul University-Cerrahpasa, Istanbul, Turkey; ^2^Department of Medical Pharmacology, Faculty of Medicine, Bolu Abant Izzet Baysal University, Bolu, Turkey; ^3^Mehmet Akif Ersoy Thoracic and Cardiovascular Surgery Training and Research Hospital, Istanbul, Turkey; ^4^Department of Medical Pharmacology, Faculty of Medicine, Biruni University, Istanbul, Turkey

**Keywords:** morphine, conditioned place preference, ropinirole, withdrawal syndrome, expression, reinstatement

## Abstract

The underlying mechanism of dependence and rewarding effects of morphine is imperative to understand. The primary aim of this study was to investigate whether ropinirole D2/3 agonist affects the rewarding and reinforcing properties of morphine-induced conditioned place preference (CPP) and withdrawal syndromes in rats. On day one, the animals were randomly divided to conduct the pre-test. The morphine (10 mg/kg, i.p.) and/or saline was administered on alternate days in an 8-day CPP session. On day 10, 15 min prior to the post-conditioning test (expression), a single dose of ropinirole (1, 2, and 5 mg/kg, i.p.) was given to rats. In extinction session, ropinirole was injected daily, and CPP was extinguished by repeated testing, with intervals of 3 days. Finally, reinstatement was assessed by administering ropinirole (1, 2, and 5 mg/kg) 15 min before the morphine injection. Morphine dependence was developed by administering increasing doses of morphine (10–50 mg/kg, i.p.). To assess withdrawal symptoms, ropinirole (1, 2, and 5 mg/kg) was injected 15 min before naloxone (2 mg/kg, s.c.) administration. The present study confirms that ropinirole attenuates expression and reinstatement of CPP, while it precipitates the extinction of morphine-induced CPP. Naloxone-precipitated morphine withdrawal symptoms, including wet dog shakes and weight loss, were attenuated although jumping was increased by a single ropinirole injection. Thus, ropinirole was influential in attenuating expression, reducing drug seeking and weakening reinstatement *via* the dopaminergic system. These findings show that ropinirole might affect neuro-adaptive changes related to dependence.

## Introduction

Adaptations within the brain reward centers and dopamine (DA) neurotransmission are involved in enhanced incentive motivation toward drug-paired stimuli, leading to drug addiction (Haleem et al., 2018). Projections of dopaminergic neurons, especially in the ventral tegmental area (VTA), have a significant role in the development of addiction (Wise and Morales, 2010; Mark et al., 2011). As for the most addictive drugs, opiates appear to exert reinforcing properties in part through their ability to increase dopamine concentration in the nucleus accumbens (Koob and Bloom, 1988). The DA system was also implicated in opiate withdrawal, and studies suggest that withdrawal is associated with reduced DA activity (Harris and Aston-Jones, 1994). For example, naloxone-precipitated withdrawal was accompanied by reductions in extracellular DA in the ventral tegmental area and the nucleus accumbens (Pothos et al., 1991; Rossetti et al., 1992). Furthermore, it was suggested that the aversive state during opiate withdrawal could be due to the suppression of DA activity (Acquas et al., 1991; Rossetti et al., 1992).

However, pharmacological studies show evidence compatible with both decreases and increases in DA activity during withdrawal, indicative of a more complex role of DA in opiate withdrawal. For example, some studies showed that DA antagonists improve (Lal et al., 1971; Lal and Numan, 1976; Levinson et al., 1994) and worsen (Harris and Aston-Jones, 1994) opiate withdrawal. Similarly, some studies showed that DA-enhancing drugs exacerbate (Ary et al., 1977; Gomaa et al., 1989) and other studies show they ameliorate (Harris and Aston-Jones, 1994) symptoms of opiate withdrawal. Other studies showed that DA agonists increase the intensity of some withdrawal symptoms and decrease that of others in the same animal (Ary et al., 1977; Ferrari and Baggio, 1982). Thus, it was suggested that some effects of opiate withdrawal may be due to increased DA activity and others to decreased activity (Ary et al., 1977).

Opioid (morphine, heroin) addiction is a chronic, relapsing/recurrent brain disease that progresses with the duration of use. Over the past few decades, the opioid crisis has attracted public attention to effective interventions for opioid addiction management (Hedegaard et al., 2017). Maintenance treatments for opioid addiction like methadone and buprenorphine are helpful as they reduce the intensity of withdrawal and craving symptoms, and naloxone is used to treat opioid overdose or opioid intoxication (Farrell et al., 1994; Connock et al., 2007; Clark et al., 2014). Though effective medications for opioid addiction are available, reinstatement and remission are still common among addicted individuals. Cravings with dreadful withdrawal symptoms increase the risk of relapse and are responsible for neurobiological changes produced by repeated abuse of opioids. Dopamine neurotransmission is important in the neurobiology of reward and aversion, although its participation in the aversive state of opioid withdrawal remains unclear.

Ropinirole is a D2/3 agonist (Perachon et al., 1999; Hoefer et al., 2006) with weak affinity to μ-opioid receptor (Tulloch, 1997). It is widely used in clinical practice for Parkinson’s disease (Francis Lam, 2000; Matheson and Spencer, 2000) and bipolar depression (Perugi et al., 2001). A pilot open-label trial of ropinirole for cocaine dependence showed promising results (Meini et al., 2008). It was also shown that ropinirole and other dopamine agonists reduce amphetamine withdrawal syndrome (Hoefer et al., 2006). The number of successful drugs available is quite limited for morphine addiction therapy. In our study, the possible effects of ropinirole in morphine addiction will be investigated.

To the best of our knowledge, ropinirole has not been the subject of any conditioned place preference or withdrawal study so far. Therefore, the primary objective of this study was to investigate the effect of ropinirole on the emergence of enhanced morphine dependence, morphine-seeking behavior, and reinstatement in rats and determine its potential effects on withdrawal symptoms. The study focused on the role of the dopaminergic system and interplay with opioid receptors.

## Materials and Methods

### Animals

Adult male Sprague–Dawley rats (obtained from Department of Laboratory Animal Biology, Institute of Experimental Medicine, Istanbul University) weighing 260–320 g were used. The animals were housed under standard laboratory conditions for at least 1 week before experimentation and given free access to food and water. All experiments were performed following the National Institutes of Health Guide for the Care and Use of Laboratory Animals and were approved by Istanbul University Local Committee on Animal Research Ethics.

### Drugs

Morphine hydrochloride was purchased from Macfarlan Smith (Edinburgh, United Kingdom). Naloxone hydrochloride and ropinirole were purchased from Sigma (St. Louis, MO. United States). All drugs were dissolved in saline to final concentrations and were applied by intraperitoneal (i.p.) or subcutaneous (s.c.) routes in 1 ml/kg volume. Control groups received saline injections at the same volume and by the same route. Drug solutions were prepared fresh immediately before each injection.

### Conditioned Place Preference

#### Apparatus

The CPP apparatus is a rectangular box (30 cm × 60 cm × 30 cm) that consists of Plexiglas. A removable Plexiglass wall divides the box into two chambers of equal size. One compartment has a black wall with a stainless-steel grid rod floor, whereas the other has a white wall with a stainless-steel mesh floor. Between the experiments for each subject, wet and dry clothes were used to clean the apparatus thoroughly.

#### Handling and Habituation

All experiments were conducted in an isolated research room with only the test subject present, between 8 a.m. and 4 p.m. Throughout the handling phase, the animals were accustomed to the hand and study room. The animals were adapted and handled in the research room to decrease stress. On the days of habituation, each rat was allowed to freely explore the apparatus for 5 min over 3 days for adaptation to the CPP apparatus.

#### Preconditioning

The day after habituation (day 1), in the preconditioning phase, the time spent in each compartment was recorded for 15 min to determine the natural place preferences of rats. The test box was considered “biased,” which means the untrained rats preferred one side (grid side) to another one (mesh side), and the rats that did not prefer any side were excluded.

During each conditioning trial, the rat had free access to the entire box with the same tactile cues and grid or mesh on both sides of the box. An animal was operationally defined as “in mesh or grid side of the box” once both forepaws were in contact with the same side. All procedures were conducted between 10:00 a.m. and 03:00 p.m. Animals were randomly assigned to morphine or saline control groups, and 8 rats were included in each group.

#### Conditioning Phase

**FIGURE 1:**
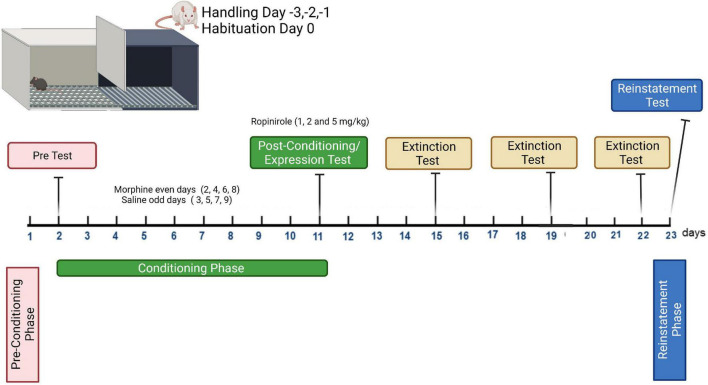
Conditioning plan of the expression, extinction, and reinstatement program for morphine-induced conditioned place preference study.

For the conditioning phase (8 days), all groups received saline (1 ml/kg) administration on the grid floor on days 3, 5, 7, and 9, and CPP was induced after pairing of morphine (10 mg/kg) with the mesh floor on days 2, 4, 6, and 8. The rats were placed in the appropriate compartment for 45 min immediately after each injection. The CPP protocol applied here conforms with previous studies involving minor modifications (Shahzadi et al., 2017; Yunusoğlu, 2021). The morphine dose was selected based on prior work showing that CPP was induced in rats at the given dose (Koo et al., 2014; Graziane et al., 2016). The protocol is shown in Figure 1.

### Conditioned Place Preference Experiments

#### Effect of Ropinirole on the Expression of Morphine-Induced Conditioned Place Preference

The day after the last conditioning day (day 10, post-conditioning), the rats were placed in the chamber with the gate removed and were allowed to freely explore the entire apparatus for 15 min. Time spent by each rat in the two chambers was recorded. To determine the effects of ropinirole on the expression of morphine-induced CPP, the rats were injected with 1, 2, and 5 mg/kg of ropinirole (i.p.) 15 min before being placed in the apparatus (test), and control groups received a saline injection (Figure 1). While there is no study about the effects of ropinirole on morphine addiction, the doses of ropinirole were chosen from somewhat similar studies (Hoefer et al., 2006; Carta et al., 2010).

#### Effect of Ropinirole on the Extinction of Morphine-Induced Conditioned Place Preference

Extinction of morphine-induced CPP was examined after the establishment of CPP in naive rats and the test was carried out every 4 days (days 14, 18, and 22) until extinction was completed in all experimental groups. During all tests, the rats were placed in the CPP device with free access to both chambers for 15 min, and the time spent in each was recorded (Mattioli et al., 2012; Yunusoğlu, 2021). The protocol is shown in Figure 1. The test was repeated for each group until the time spent in the morphine-paired compartment of experimental groups became insignificant compared to that of control. To determine the effects of ropinirole on the extinction of morphine-induced CPP the rats were injected, once a day, with 1, 2, and 5 mg/kg of ropinirole in the home cage, morphine and control groups received a saline injection.

#### Effect of Ropinirole on the Morphine-Primed Reinstatement of Conditioned Place Preference

The reinstatement procedure was performed after extinction was completed in all experimental groups. Extinction of morphine-induced CPP was reinstated by priming injections of morphine at a dose of 2 mg/kg i.p. One day after the last extinction trial (day 23), rats that received ropinirole (1, 2 and 5 mg/kg, i.p.) or saline, 15 min before a priming injection of morphine (2 mg/kg, i.p.), were immediately tested for reinstatement of CPP (Figure 1). During this reinstatement test, the rats were permitted free access to the entire CPP chamber for 15 min, and the time spent in each chamber was measured (Mattioli et al., 2012; Yunusoğlu, 2021).

### Induction of Dependence and Measurement of Withdrawal Signs

#### Induction of Morphine Dependence

Morphine dependence was induced in randomly chosen rats. Injection of morphine (10 mg/kg, i.p.) twice a day at 12 h interval (08:00 a.m./p.m.) was applied by increasing doses by 10 mg/kg each day for 5 days, and 50 mg/kg morphine was injected once in the morning on day 6. Withdrawal signs were precipitated with naloxone (2 mg/kg, s.c.) 4 hours after the last morphine injection. To determine the effects of ropinirole on the withdrawal signs precipitated with naloxone, the rats were injected with different doses of ropinirole (1, 2 and 5 mg/kg, i.p.) 15 min before injection of naloxone. Control groups received saline. The doses of morphine (10–50 mg/kg, i.p.) and naloxone (2 mg/kg, s.c.) were chosen from a previous study by one of our colleagues (Allahverdiyev et al., 2011) and a similar study (Zarrindast et al., 2003) with minor modifications.

#### Measurement of Naloxone-Precipitated Withdrawal Signs

An observer or investigator who was unaware of the treatment protocol evaluated the experiments. After injection of naloxone, the rats were placed individually into observation cages. Signs of naloxone-precipitated withdrawal were measured in a quiet room. Wet-dog shakes and jumping were counted as signs of withdrawal for 15 min. The rats were weighed before and after the experiment to measure withdrawal-induced weight loss.

### Statistical Analysis

The data are expressed as the means ± *SD* and were analyzed by GraphPad Prism ^®^ (Version 5.0; GraphPad Software, Inc., La Jolla, CA, United States) software. Unpaired Student’s *t*-test was used for pairwise comparison of compartments (pretest). The effects of ropinirole on the expression and extinction of morphine-induced CPP were evaluated with two-way analysis of variance (ANOVA) for repeated measurements (treatment × days) and Tukey test was used as *post hoc* analyses. One-way ANOVA was used to determine the effect of different doses of ropinirole on reinstatement of morphine-induced CPP and naloxone-precipitated withdrawal signs. The Dunnett test was used for the *post hoc* analysis. The level of statistical significance was set at *p* < 0.05.

## Results

### Place Preferences of Rats in the Preconditioning Phase

**FIGURE 2:**
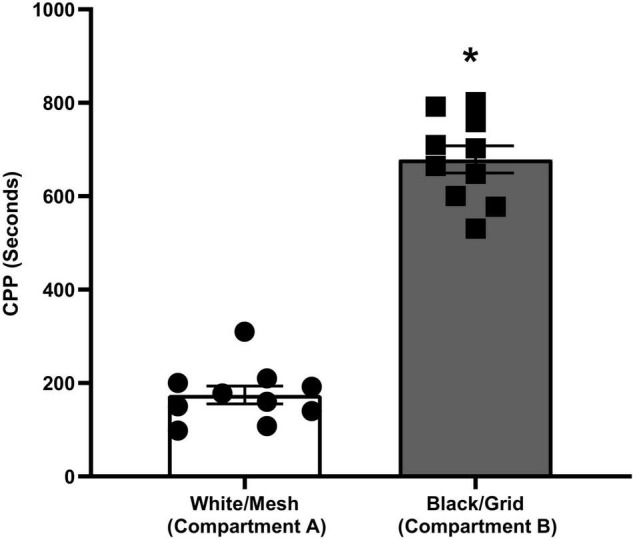
Conditioned place preference of rats during pretesting (*p < 0.001, Student’s *t*-test).

The place preferences of the animals between compartments A and B on the pretest day are given in Figure 2. During the pretest trial, animals spent significantly (*p* < 0.001) more time on the grid floor than on the mesh floor. The preference made by rats clearly indicates that the apparatus is biased.

### Effect of Ropinirole on the Expression of Morphine-Induced Conditioned Place Preference

**FIGURE 3:**
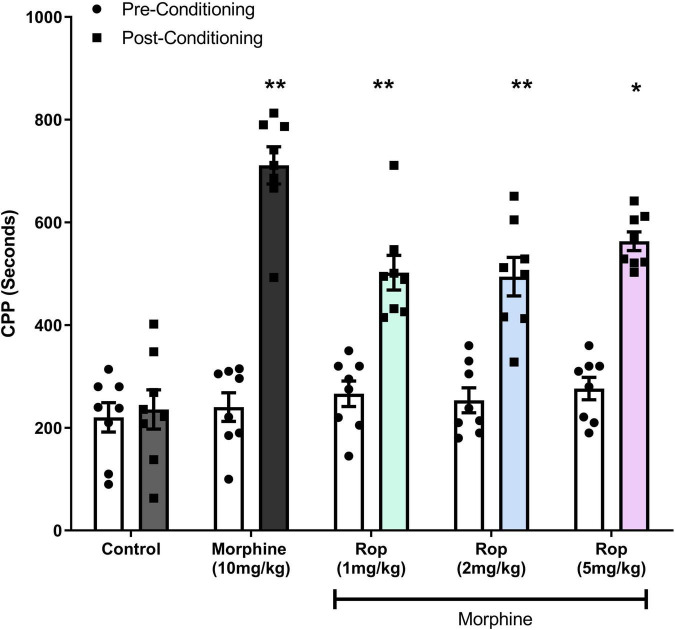
Effect of combination treatment on morphine-induced CPP (combinations: 1, 2, and 5 mg/kg Rop plus 10 mg/kg morphine). Data expressed as mean ± SD (*n* = 8). **p < 0.01, *p < 0.05 relative to the preconditioning group; Tukey test. Ropinirole-Rop.

The effects of ropinirole on the expression of morphine-induced CPP is shown in Figure 3. Two-way ANOVA test indicated that pre- and post-conditioning morphine treatment had a significant main effect on CPP [*F*(1, 7) = 138.3; *p* < 0.001]. A significant main effect was detected for dosage groups [*F*(4, 28) = 19.05; *p* < 0.001]. There was also an interaction between pre–post conditioning tests (days) and drug doses [*F*(4, 28) = 18.24; *p* < 0.001]. *Post hoc* analysis with the Tukey’s test revealed that morphine (10 mg/kg) produced statistically significant CPP in rats (*p* < 0.001), similarly combination (Rop 1, 2, and 5 mg/kg plus morphine 10 mg/kg) groups significantly decreased the morphine-induced CPP (*p*s < 0.05).

### Effect of Ropinirole on the Extinction of Morphine-Induced Conditioned Place Preference

**FIGURE 4:**
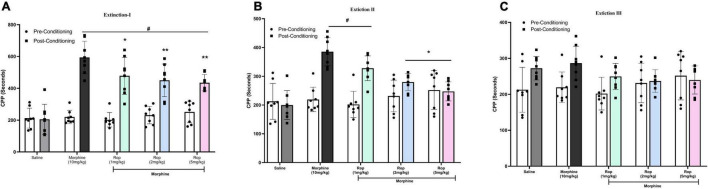
The effect of ropinirole on extinction of morphine-induced CPP. Rats were injected with either saline/morphine (for saline and morphine control groups) or ropinirole (1, 2, and5 mg/kg) in periods of three days after the post-conditioning test. Data expressed as mean ± SD (*n* = 8) **(A)** Extinction I (day 14), ^#^*p* < 0.001 compared with saline post-conditioning group and *p < 0.05, **p < 0.01 compared with the morphine post-conditioning group. **(B)** Extinction II (day 18), ^#^*p* < 0.05 relative to the saline post-conditioning group and *p < 0.01 compared with the morphine post-conditioning group. **(C)** Extinction III (day 22) all the groups were non-significant. Ropinirole-Rop.

Following the post-conditioning phase, repeated ropinirole or saline injections were applied once a day until extinction was completed in all experimental groups. The test was repeated every 4 days (days 14, 18, and 22) for each group until the time spent in the morphine-paired compartment by the experimental groups was similar to that of the saline control group. After the establishment of adequate CPP in rats from all morphine-treated groups (*p* < 0.001), the effect of ropinirole on the extinction of morphine-induced CPP was studied. Repeated measures two way-ANOVA revealed that ropinirole augmented CPP extinction (Extinction III) as shown in Figure 4. Extinction I [Effect of treatment: *F* (4, 28) = 12.93, *p* < 0.001; effect of days: *F*(1, 7) = 171.7, *p* < 0.001; treatment x days: *F*(4, 28) = 13.50, *p* < 0.001], Extinction II [Effect of treatment: *F*(4, 28) = 9.51, *p* < 0.001; effect of days: *F*(1,7) = 35.83, *p* < 0.001; treatment x Days: *F*(4, 28) = 8.72, *p* < 0.001] and Extinction III [Effect of treatment: *F*(4, 28) = 0.5885, *p* > 0.05; effect of days *F*(1, 7) = 11.8, *p* < 0.01; treatment x Days: *F*(4, 28) = 3.659, *p* < 0.01].

### Effect of Ropinirole on the Morphine-Primed Reinstatement of Conditioned Place Preference

**FIGURE 5:**
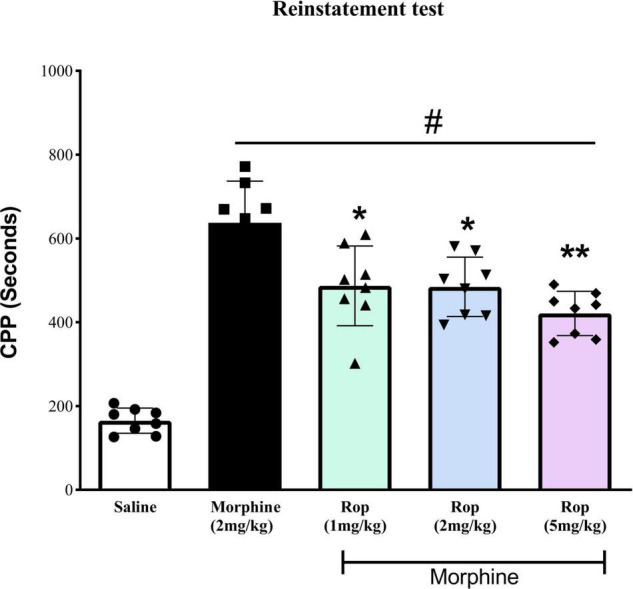
The effect of ropinirole drug-priming reinstatement of morphine-induced CPP. After the last extinction test rats received different doses of ropinirole (1, 2, and 5 mg/kg) or saline 15 min prior to the priming injection of morphine (2 mg/kg) and were tested for the reinstatement of CPP. Results represent mean ± *SD* (*n* = 8). ^#^*p* < 0.001 compared with saline control group and *p < 0.01, **p < 0.001 compared with the morphine group. Ropinirole-Rop.

To evaluate whether the extinction of CPP was established in the animals, repeated measures one-way ANOVA was carried out on the pre-CPP, extinction and saline-primed groups. The results showed a main significant effect on CPP [*F*(4, 35) = 42.95, *p* < 0.001]. Tukeys’s test indicated that the extinguished CPP was completely reinstated after the administration of a single low dose of morphine (2 mg/kg) compared with saline-paired chamber (*p* < 0.001; Figure 5). This showed that the CPP model was successfully established. Pretreatment with ropinirole 1 mg/kg (*p* < 0.01), 2 mg/kg (*p* < 0.01) and 5 mg/kg (*p* < 0.001) attenuated the effect of morphine on reinstatement compared to the morphine-paired chambers (Figure 5).

### Effects of Ropinirole on Naloxone-Precipitated Withdrawal in Morphine-Dependent Rats

**FIGURE 6:**
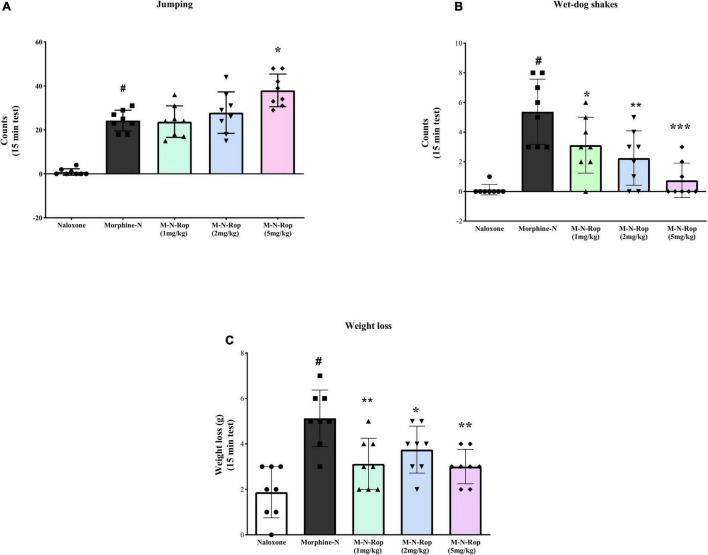
The effect of ropinirole on morphine withdrawal syndrome. Results represent mean ± *SD*, one-way ANOVA *post hoc* Dunnett’s test (*n* = 8). **(A)** jumping count; ^#^*p* < 0.001 compared with naloxone group and *p < 0.01 compared with the morphine-naloxone (Morphine-N) group. **(B)** wet-dog shakes count; ^#^*p* < 0.001 compared with naloxone group and *p < 0.05, **p < 0.01, ***p < 0.001 compared with the morphine-naloxone (Morphine-N) group. **(C)** weight loss; ^#^*p* < 0.001 compared with naloxone group and *p < 0.05, **p < 0.01, **p < 0.01 compared with the morphine-naloxone (Morphine-N) group. Morphine-M, Naloxone-N, Ropinirole-Rop.

Withdrawal symptoms were present significantly in rats given naloxone following repeated morphine injections. As shown in Figure 6, one-way ANOVA revealed significant differences in jumping counts [*F*(4, 35) = 33.68, *p* < 0.001], wet-dog shakes count [*F*(4, 35) = 13.05, *p* < 0.001] and weight loss [*F*(4, 35) = 9.86, *p* < 0.001]. *Post hoc* Dunnett test revealed that naloxone significantly increased jumping, wet-dog shakes, and weight loss (*p* < 0.001). Ropinirole (5 mg/kg) significantly increased naloxone-precipitated jumping in morphine-dependent rats (*p* < 0.01; Figure 6A), while other doses of ropinirole did not cause any change. On the other hand, in Figure 6B, ropinirole (1, 2, and 5 mg/kg) significantly decreased wet-dog shakes (*p* < 0.05, *p* < 0.01, *p* < 0.001, respectively. Similarly, Ropinirole (1, 2, and 5 mg/kg) significantly decreased naloxone-precipitated weight loss in morphine-dependent rats (*p* < 0.01, *p* < 0.05, *p* < 0.01, respectively; Figure 6C).

## Discussion

Studying and exploring the addiction potential of morphine is very important for many reasons, firstly for its frequent use as a potent analgesic in metastatic patients and secondly for its potential for abuse, a heavy burden on healthcare systems.

The purpose of the present study was to demonstrate whether or not ropinirole attenuates morphine-induced CPP and the symptoms of naloxone-precipitated morphine withdrawal syndrome. The main findings of the current study revealed that (i) ropinirole decreased priming-induced reinstatement of morphine CPP, (ii) ropinirole attenuated expression of morphine-induced CPP, and (iii) the D2/3 selective receptor agonist ropinirole decreased extinction of morphine CPP.

Scientists have always remained curious about the addiction potential of the most potent analgesic agent. Nevertheless, in spite of some definitive studies enriching the literature (Wu et al., 2019; Zeng et al., 2020); no clinical or animal studies examined the ropinirole effect on morphine addiction. Morphine dependence alters behavior by affecting dopamine receptor signaling; decreased receptor activation contributes to withdrawal syndrome, and low dopamine levels in brain reward centers play a significant role in opioid withdrawal (Adinoff, 2004). This study investigated the possible effects of the selective dopamine receptor agonist ropinirole on morphine-induced CPP. For this, two separate experimental protocols were developed for evaluation in terms of psychic and physical dependence.

The first experimental findings showed that ropinirole decreased the expression, extinction, and reinstatement of morphine-induced CPP. Morphine/opioid μ receptors are densely located in the ventral tegmental area, one of the important primary reward centers. Activation of these receptors stimulates the mesocorticolimbic dopaminergic system and significantly increases the dopamine level (Kalivas, 1993; Olmstead and Franklin, 1997). D1 and D2 receptors have an essential role in reward-related learning in the conditioned place preference paradigm (Ranaldi and Beninger, 1993; Beninger, 1998). Addictive behavior depends strongly on mesolimbocortical dopaminergic responses; repetitive behaviors were associated with dopaminergic dysregulation in the basal ganglia–thalamo–cortical circuitry (Schmidt et al., 2013). The literature strongly supports the role of D2 receptors in dependence; however, the role of D1 receptors is somewhat undefined. A D1 receptor agonist produced conditioned place preference when given to the nucleus accumbens, one of the vital reward centers (White et al., 1991); however, systemic application leads to conditioned place aversion (CPA). In another study, chronic administration of a D2 receptor agonist quinpirole was shown to enhance the reward-facilitating effects of amphetamine with respect to responding to intracranial self-stimulation (Schmidt et al., 2013). Hippocampal dopamine D2 receptor activity is positively related to working memory performance (Wilkerson and Levin, 1999), and the involvement of ventral hippocampal D2 receptors in memory performance was proposed (Umegaki et al., 2001).

To the best of our knowledge, the role of ropinirole in learning or reward behavior treatment has been the subject of very few investigations. A recent study showed that chronic ropinirole treatment led to a pattern of changes and upregulation of the β-arrestin-AKT-GSK3β intracellular cascade in compulsive-like gambling behavior. It was recently theorized to dominate D2-mediated signaling under hyperdopaminergic conditions in the dorsal striatum (Cocker et al., 2016).

Morphine-induced CPP reduced once a D3 receptor agonist was given prior to testing (Kalivas, 1993). On the other hand, a D1 receptor agonist prevented the development of morphine dependence (Zarrindast et al., 2003). In a similar study, the D3 receptor agonist 7-OH-DPAT prevented the acquisition and expression of morphine dependence (De Fonseca et al., 1995).

In the second experiment, we investigated the effects of ropinirole on morphine physical dependence. The withdrawal signs were precipitated by naloxone following repeated morphine injections and the rats were injected with different doses of ropinirole (1, 2, or 5 mg/kg, i.p.) 15 min prior to naloxone. In a dose-dependent manner, ropinirole decreased the weight and signs of wet dog shakes; however, it increased jumping.

The dopaminergic system has a significant role in opioid addiction (Smith et al., 1985) and opioid withdrawal symptoms (Koob and Bloom, 1988; Harris and Aston-Jones, 1994). Many studies showed that mesolimbic DA activity decreases in opioid withdrawal (Rossetti et al., 1992; Harris and Aston-Jones, 1994). Administration of the dopamine receptor antagonist NAc produced withdrawal symptoms similar to opioid withdrawal. The systemically administered dopamine agonist apomorphine significantly reduced opioid withdrawal (Harris and Aston-Jones, 1994). At the same time, chronic opioid administration decreases dopaminergic sensitivity (Nestby et al., 1995; Walters, 2000). It was shown that naloxone-induced withdrawal symptoms increase with D2 antagonist administration. Nevertheless, this effect disappears with the D2 receptor agonist (Harris and Aston-Jones, 1994). The literature supports the role of dopamine receptor agonists in relieving withdrawal symptoms though differentially (Ohmura et al., 2011). Prefrontocortical monoaminergic changes play a role in the behavioral expression of opiate withdrawal; the severity of some withdrawal signs is related to the dopaminergic and serotonergic tone of the medial prefrontal cortex. Additionally, an inverse relationship exists between mesocortical and mesolimbic dopaminergic systems (Espejo, 2001).

Gomaa et al. (1989) showed that dopamine 2 agonist bromocriptine exacerbated opiate withdrawal signs like jumping in morphine-dependent mice. In another study administration of apomorphine before naloxone significantly decreased the naloxone ED50 for inducing withdrawal jumping in mice that were pretreated with morphine (Martin and Takemori, 1987). Many studies support that both naloxone-induced withdrawal and spontaneous opioid withdrawal symptoms decrease the dopamine level in brain reward centers (Acquas and Chiara, 1994; Crippens and Robinson, 1994; Shaham et al., 1996). Further experiments are required to explore the exact underlying mechanism involved in increased jumping.

These somatic signs are important as it is accepted that affective drug withdrawal symptoms are of major motivational significance in reinstatement and continued drug use. The literature suggested that morphine withdrawal associated with negative affective states and place aversion to previous neutral environmental stimuli could represent a motivational component in maintaining drug abuse (Budzynska et al., 2012). Thus, it is important to understand the mechanisms that mediate affective behaviors during morphine withdrawal.

This research has some limitations that must be noted when interpreting the conclusions. This experiment was conducted on male rats. Gender differences were observed in numerous aspects of the pharmacology of opioids, including a higher sensitivity to antinociceptive properties (Cook et al., 2000), discriminative stimulus effects (Craft and Stratmann, 1996), and their ability to generate physical dependence in male rats (Cicero et al., 2000), as well as enhanced responsiveness in females to reinforcing properties by conditioned place preference and self-administration methods (Cicero et al., 2000, 2003). Sex differences in reinforcing and substance utilization found that dose-related dependence in females commences at lower doses than males (Karami and Zarrindast, 2008; Becker and Koob, 2016; Díaz-Mesa et al., 2016). This fact is an issue for future research to explore.

## Conclusion

To the best of our knowledge, in this study for the first time, we found that the selective dopamine agonist ropinirole reduces the expression, reinstatement, and morphine withdrawal symptoms and accelerates the extinction of morphine CPP. These data show that ropinirole may be helpful in the treatment of addiction and withdrawal symptoms of morphine and other opiate agents.

## Data Availability Statement

The raw data supporting the conclusions of this article will be made available by the authors, without undue reservation.

## Ethics Statement

The animal study was reviewed and approved by the Ethics Committee of the Department of Laboratory Animals Science, Aziz Sancar Institute of Experimental Medicine, Istanbul University.

## Author Contributions

AS conceived, designed, and did statistical analysis and manuscript write-up. OY and EK performed the experiment and manuscript editing. HS did manuscript editing and statistical analysis. ZY contributed to design, review, and final approval of manuscript. All authors contributed to the article and approved the submitted version.

## Conflict of Interest

The authors declare that the research was conducted in the absence of any commercial or financial relationships that could be construed as a potential conflict of interest.

## Publisher’s Note

All claims expressed in this article are solely those of the authors and do not necessarily represent those of their affiliated organizations, or those of the publisher, the editors and the reviewers. Any product that may be evaluated in this article, or claim that may be made by its manufacturer, is not guaranteed or endorsed by the publisher.
